# Fault Diagnosis and Fault Frequency Determination of Permanent Magnet Synchronous Motor Based on Deep Learning

**DOI:** 10.3390/s21113608

**Published:** 2021-05-22

**Authors:** Chiao-Sheng Wang, I-Hsi Kao, Jau-Woei Perng

**Affiliations:** Department of Mechanical and Electro-Mechanical Engineering, National Sun Yat-sen University, Kaohsiung 804, Taiwan; d093020010@nsysu.edu.tw (C.-S.W.); ihkao@berkeley.edu (I.-H.K.)

**Keywords:** class feature map, convolutional neural network, deep learning, fault diagnosis, permanent magnet synchronous motor

## Abstract

The early diagnosis of a motor is important. Many researchers have used deep learning to diagnose motor applications. This paper proposes a one-dimensional convolutional neural network for the diagnosis of permanent magnet synchronous motors. The one-dimensional convolutional neural network model is weakly supervised and consists of multiple convolutional feature-extraction modules. Through the analysis of the torque and current signals of the motors, the motors can be diagnosed under a wide range of speeds, variable loads, and eccentricity effects. The advantage of the proposed method is that the feature-extraction modules can extract multiscale features from complex conditions. The number of training parameters was reduced so as to solve the overfitting problem. Furthermore, the class feature map was proposed to automatically determine the frequency component that contributes to the classification using the weak learning method. The experimental results reveal that the proposed model can effectively diagnose three different motor states—healthy state, demagnetization fault state, and bearing fault state. In addition, the model can detect eccentric effects. By combining the current and torque features, the classification accuracy of the proposed model is up to 98.85%, which is higher than that of classical machine-learning methods such as the k-nearest neighbor and support vector machine.

## 1. Introduction

As industrial automation becomes increasingly popular, motors are used in various mechanical systems to supply power. The advantage of automation is that it makes production lines faster and more flexible. Faults in the motors and machine elements, including bearings, gearboxes, and shafts, may result in substantial financial costs and human safety problems. Early fault diagnosis and detection are essential for maintaining the high performance and reliability of the entire mechanical system [1]. Fault diagnosis can prevent unexpected lengthy process shutdowns, damage to the mechanical system, unnecessary maintenance operations, and even expensive repairs. Therefore, to prevent catastrophic motor failure, early fault diagnosis of the motor and machine elements is important.

Generally, motor fault diagnosis is divided into mechanical and electrical faults [2,3]. Mechanical faults include air-gap deformation, bearing failures, shaft misalignment, and mechanical imbalance, as presented in the literature [4,5,6]. Furthermore, electrical faults usually include the stator, rotor, and electrical supply faults, which were analyzed in [7,8,9]. Various diagnostic techniques for mechanical systems have been presented in the literature [10,11,12]. Thermal image and acoustic-based methods both have the advantage of being non-invasive. In [13], the feature areas of a thermal image were determined by calculating the difference between the thermal images. After finding the areas, the images were converted into binary images for fault classification. However, the method based on the thermal image has a disadvantage in that the machine is damaged while collecting the dataset over a long time under high-temperature conditions. The acoustic-based technique was analyzed in [14,15]. It has a lower cost than the thermal-based method. Generally, defects in the motor are identified by analyzing the features of the frequency spectrum of the sound emission. However, in real conditions, the acoustic signal is easily mixed with other signals and interferes with environmental factors. Many mechanical fault diagnosis applications based on vibration signal analysis are also available, especially for bearing faults [16] and gear transmission systems [17]. In [18], 12 accelerometers were simultaneously used. Moreover, the time and frequency domains of the vibration signal were analyzed separately to determine the failed components of the gearbox. Article [19] presents a time–frequency analysis of a vibration signal that solves time-varying faults under variable speed conditions. However, the position at which the accelerometer is set is often challenging.

Motor current signature analysis (MCSA) is a fault diagnosis technique based on motor current analysis. MCSA is one of the most widely used fault diagnosis techniques for motors, and has the advantage of simplicity of current sensors and installation. The fast Fourier transform (FFT) is a well-known method that computes the discrete Fourier transform of a discrete-time series function. The FFT method produces computationally efficient results; hence, it is a powerful and simple MCSA technique. In a previous study [20], the motor current signal under a transient working condition was analyzed via the discrete wavelet decomposition for a gearbox faults detection. In another study [21], multiple current sensors were used to diagnose the gearbox faults. The frequency domain signals transferred by FFT are stacked to create a matrix as an input vector of the 2D convolutional neural network (CNN). 

Machine learning has become a popular technique. In general, machine learning can be classified into supervised and unsupervised learning. Unsupervised learning is a training method that does not require any labels, such as the principal component analysis [22], k-nearest neighbor (KNN) algorithm [23], and generative adversarial networks [24]. Supervised learning requires a correct label for the training model, such as the support vector machine (SVM) [25], artificial neural networks [26], and linear regression [27]. Many studies have shown that machine learning can effectively solve problems associated with motor fault diagnosis [28].

Recently, the fast-growing deep learning algorithm, a part of machine learning, has been widely used in this field. In the literature [29], two motor fault diagnosis methods were proposed to detect five motor conditions. The motor conditions include a normal permanent magnet synchronous motor (PMSM), two different degrees of demagnetization fault PMSMs, a bearing with a damaged inner ring, and a bearing with aluminum powder. The fault diagnosis technique effectively detected the five conditions over a wide speed range. After data acquisition of the stator current, the discrete wavelet transform (DWT) was utilized to extract the features. The softmax classifier classified the approximation and detail coefficients obtained through the transformation. To achieve a higher accuracy and a reliable fault-detection method, a 1D CNN was proposed. By stacking the convolutional, max-pooling, and batch normalization layers, the 1D CNN automatically learned the important features from the time-domain signal. The final classification accuracy was up to 98.8%, which was 0.7% higher than that of the DWT method.

However, in real conditions, most motors are operated under loads, which is not considered in [29]. To obtain robust and reliable motor fault diagnosis and detect several fault conditions simultaneously, motor current and vibration signals were leveraged together in [30]. Both the motor current and vibration signals were converted into a time–frequency distribution using the wavelet transform. The time–frequency distributions were treated as grayscale images, which were sent to the multi-signal 2D CNN. Two architectures of the 2D CNN with similar parameters were discussed: a model that takes a two-channel signal as the input vector and the other that takes two individual signals as the input vectors. These results indicate that the latter model had a higher accuracy rate. In [31], a stacked inverted residual CNN (SIRCNN), which is a lightweight model, is proposed to diagnose rolling bearing faults. The time domain vibration signal is transformed into a 2D image after normalization. By using the depth-wise separable convolution, linear bottleneck, and inverted residual block, the computations and size of the model can be decreased. Moreover, the authors of [31] indicate that SIRCNN is highly robust against different noisy environments, with the addition of white Gaussian noise to the original signal. In a previous study [32], a bearing defect diagnosis model was trained based on the transfer learning methodology. The model was first trained by the source domain data; next, the samples of the target domain were used to fine-tune the mode. Furthermore, in another study [32], a novel trigonometric cross-entropy function calculating the sparsity cost was developed and included in the cost function. The modified cost function can evade the redundant activation of neurons in the hidden layer. A previous study [33], with different results than those of [30], proposed a multiresolution multisensory fusion network consisting of a 1D CNN and long short-term memory (LSTM). By combining the 1D CNN and LSTM, the model can learn features from a two-channel signal well. Moreover, the authors of [33] highlight the effectiveness of multiple kernels in finding different scales of features. The power supply frequency was considered and eliminated using the Hilbert transform. Furthermore, in contrast with other studies, one [33] used three load conditions, which were closer to real applications.

In this paper, a 1D CNN, which is a weakly supervised learning model, is proposed. The 1D CNN model consists of multiscale feature-extraction modules and has the ability to automatically determine the localization of the frequency component contributes to the classification. The experimental results demonstrate that the proposed method can effectively diagnose three different motor states running at variable speeds, load conditions, and eccentric effects. The results of the proposed 1D CNN were better than those of the previous methods. Therefore, we recommend the use of the proposed 1D CNN for feature-extraction and the softmax layer for classification for higher classification accuracy.

The contributions of this paper are as follows:

(1) Unlike the aforementioned related studies, the 1D CNN model proposed in this paper diagnoses motor faults by extracting the stator current signal and torque signal of the motor.
(2) In [33], a multilevel information fusion model, combined with a 1D CNN and LSTM, was used to diagnose the motor faults. The model can detect five different motor faults by extracting the vibration and stator current signals. However, Wang et al. [33] considered only three fixed load settings. This study considered variable loads ranging from 0 to 0.24 Nm.
(3) The parameters and sizes of the neural network can be reduced using the proposed feature-extraction module. Furthermore, the model remains robust and can obtain a high classification accuracy.
(4) In the aforementioned studies, the exact frequency of the signal contributing to the classification was not shown. This study implemented a weakly supervised architecture and visualized the important grades of the frequency components that contribute to the classification.
(5) In summary, we propose a 1D CNN model to detect motor faults, under a wide range of motor speeds from 100 to 1600 rpm and loads from 0 to 0.24 Nm. In addition, the model can detect the effect of eccentricity and identify important frequency components.

The remainder of this paper is organized as follows. Section 2 presents the design of the motor diagnosis platform. Section 3 describes the structure of the diagnosis system and explains the diagnosis steps. In Section 4, the experimental results obtained from real-time motor data, which demonstrate the effectiveness and robustness of the proposed methods for motor condition monitoring, are presented. The experimental results show that the proposed 1D CNN model can effectively classify. Section 5 presents the conclusions of this study.

## 2. Motor Diagnosis Platform and Sensors

**Figure 1:**
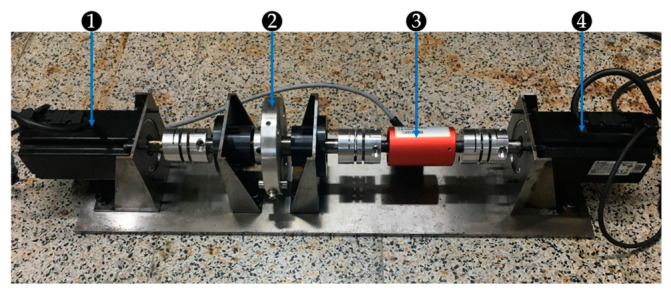
Design of the motor fault diagnosis platform: (1) testing motor, (2) load disk, (3) torque sensor, and (4) load motor.

The motor diagnosis platform built in this study is shown in Figure 1. The critical peripheral devices of the platform include two PMSMs, one torque sensor, and one Hall sensor. The PMSM used was ECM-A3L-0807, which was manufactured by Delta Electronics. The maximum rpm and torque of the motor were 3000 rpm and 2.39 Nm, respectively. The torque sensor used was DATAFLEX^®^ 16, which was developed by KTR Systems GmbH. The torque measurement error was 0.1% with angular, radial, and axial offset compensation performances. Furthermore, the torque sensor had a maximum torque measurement of 16 Nm. The Hall sensor, ACS711EX, could handle bidirectional currents from −31 to +31 A with a 100 kHz bandwidth. The data acquisition unit was a USB-2405, developed by ADLINK Technology. It used the 24-bit Sigma-Delta ADC with a built-in anti-aliasing filter and four simultaneous sampling analog input channels up to 128 kS/s.

**Figure 2:**
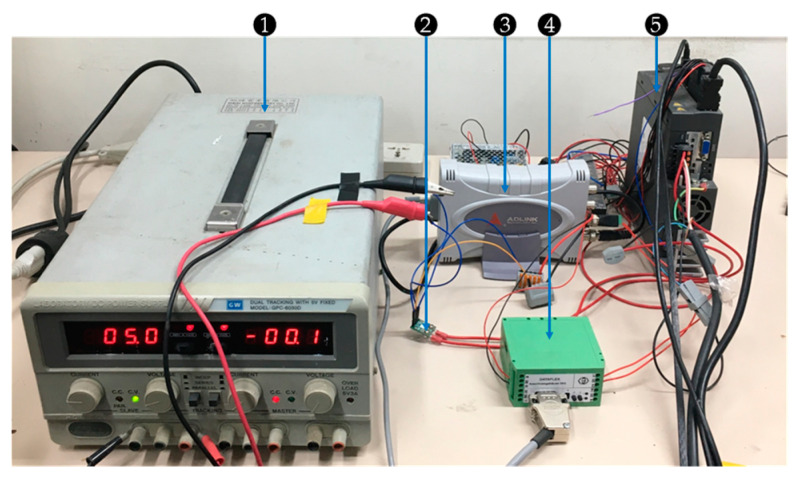
Peripheral devices: (1) power supplier, (2) hall sensor, (3) USB-2405, (4) signal amplifier of the torque sensor, and (5) actuator.

Figure 1 shows the two motors on the platform. The motor on the left side was the power source of the mechanical system. This motor was the testing motor, which was diagnosed while it rotated. Three types of testing motors were analyzed: a healthy motor, a motor with demagnetization failure, and a motor with bearing fault. The motor on the right side was the load motor, which provided inverse torques to the testing motor. The actuator controlled the torque of the load motor. A torque sensor was installed between the testing and load motors to measure the torque value of the load motor. Figure 2 illustrates the peripheral devices. The power supplier was used to provide a power of 5 V to the Hall sensor. The Hall sensor was clamped to the testing motor using one of the three-phase wires for the current signal acquisition. A load disk with four holes was placed on the axis. The eccentric effect could be generated by locking a small weight on one of the holes while the motor was rotating.

## 3. Methods

In this section, the proposed motor fault diagnosis techniques are described. First, the stator current and torque signals were collected under several conditions, and the dataset was introduced. To make the data more suitable for the proposed 1D CNN model, the dataset was processed first. Then, the feature-extraction module and 1D CNN were designed. A diagnosis model design process was utilized to confirm the reliability and classification accuracy rate of the proposed 1D CNN model. After the experiment was tested using a real motor diagnosis platform, the hyperparameters of the 1D CNN model were obtained, as shown in this section. The performance of the model and the associated frequency automatically determined by the proposed 1D CNN model are discussed in Section 4.

**Figure 3:**
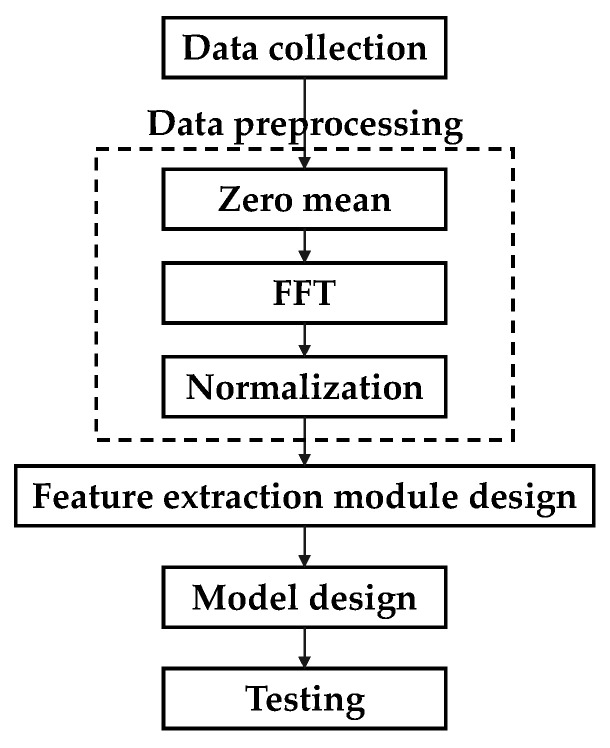
Flowchart of the proposed method.

The flowchart of the proposed method is shown in Figure 3. 

### 3.1. Data Collection and Signal Preprocessing

#### 3.1.1. Data Collection

Experiments for the motor fault diagnosis were conducted on the motor fault diagnosis platform, as illustrated in Table 1. The stator current and torque signals were simultaneously collected using a USB-2405 at a sampling rate of 12,800 Hz. The motor types included a healthy motor, a demagnetized motor, and a motor with bearing fault. Two of the loading conditions were fixed at 0 and 0.24 Nm. To ensure that the model could diagnose motors while the loads were changing, the data on loads were collected while the loads changed randomly in the range of 0–0.24 Nm. Because of the actuator setting, the discrete torque commands were represented as $T_{c}=\left[0,\;0.024,\;\ldots ,\;0.216,\;0.24\right]$. The actuator continuously and randomly chose one torque value in $T_{c}$, and sent a step torque command to the motor. Furthermore, the frequency of the varying load was 10 Hz. The detection range of the operating speed was from 100 to 1600 rpm, and the data were collected every 100 rpm. The third operating condition was affected by the load disk. The eccentricity could be controlled by locking or unlocking the weight on the load disk. 

For every motor condition, 500 measurements were obtained. Hence, the total number of datasets was 144,000 (3×3×16×2×500), indicating three failure modes, three loading conditions, 16 rotating speeds, 2 eccentric modes, and 500 measurements. The proposed model had two output layers: one output was the classification of the failure mode of the motor, and the other was the detection of the eccentric effect. Therefore, the samples were labeled according to the failure mode of the motor and the eccentricity of the load disk for supervised learning. In this study, the ratio of training data to testing data was 4:1. To ensure that the model learned the features from every condition, the data were divided equally based on the operating conditions.

#### 3.1.2. Signal Preprocessing

The raw torque and current signals collected were a 1D time series. To eliminate the DC voltage from the power supplier, the raw data, $S_{r,k}=\left[S_{r,0},\cdots ,S_{r,l-1}\right]$, went through the zero-mean operation first, as follows:

(1) $$S_{z,k}=S_{r,k}-\frac{\sum_{n=0}^{l-1}S_{r,n}}{l},\;k=0,\ldots ,l-1,$$

where $S_{z}$ is the signal after zero-mean operation. $S_{r}$ is the raw signal, and l is the length of the raw signal. Then, the signal was converted from the time domain to the frequency domain using the FFT method. The FFT calculation is as follows:

(2) $$S_{f,k}=\sum_{n=0}^{l-1}S_{z,n}e^{-i2\pi k\frac{n}{l}},\;k=0,\ldots l-1,$$

where $S_{f}$ is the transformed signal, and only the amplitude of the frequency signal is analyzed. Subsequently, the frequency signal was normalized into the range of [0,1] using the following expression:

(3) $$S_{N,k}=\frac{\left|S_{f,k}\right|}{Max\left(\left|S_{f}\right|\right)},\;k=0,\ldots ,l-1,$$

where $S_{N}$ is the normalized signal and $Max\left(\left|S_{f}\right|\right)$ represents the maximum amplitude of the frequency components. The normalization operation has two advantages: (1) it is convenient to observe the signal difference between different failure modes, and (2) it helps the model converge faster while training the deep learning model.

### 3.2. Feature-Extraction Module

**Figure 4:**
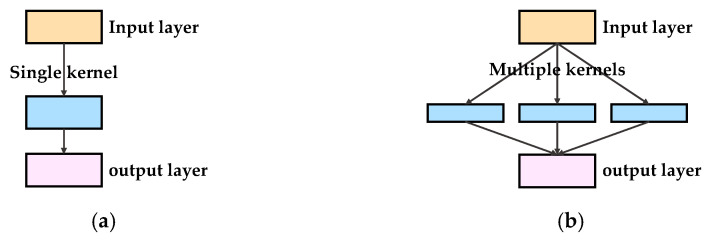
Convolutional architecture: (**a**) densely connected and (**b**) sparsely connected.

As mentioned in Section 3.1.1, three motor failure modes, namely, variable rotating speeds of the motor, the loading effect, and the eccentric effect, were analyzed. The operating conditions were very complex for motor diagnosis. The features of the motor varied substantially in terms of location and size. The fixed kernel size used in the traditional CNN model was changed. A larger kernel size of the convolutional layer was preferred in order to obtain global information, whereas the smaller one obtained the local information. To allow the model to learn from the complex motor conditions, a sparsely connected network architecture was used instead of a densely connected architecture. Figure 4 illustrates the difference between the two convolutional architectures. Through the application of multiple convolution filters, as shown in Figure 4b, the network learned the multilevel features from the same input.

In addition to the sparsely connected convolutional layer, a 1×1 convolution was used in the proposed neural network. The main purpose of using a 1×1 convolution was to control the dimensionality of the convolutional layer [34,35]. Furthermore, by adding a 1×1 convolution with a nonlinear activation function, the nonlinearity enabled the model to learn more complex functions. In [36,37], the residual connection was successfully used in the ResNet and Inception neural networks. The residual connection [38] helped the model prevent vanishing gradients. By adding the identity input, the convolutional layer enabled the learning of different features. In [31], the residuals were used in the stacked inverted residual convolution neural network model for bearing fault diagnosis. Through the implementation of stacks of inverted residual blocks, the model fulfilled a lightweight design and maintained a fast and highly accurate diagnosis.

**Figure 5:**
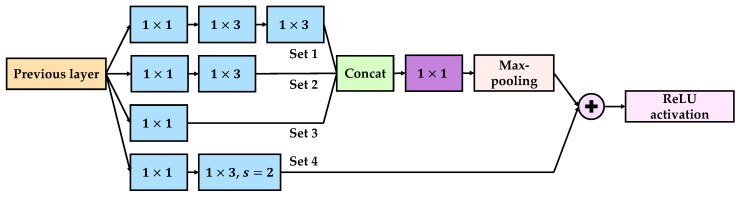
Structure of the proposed feature-extraction model.

The structure of the proposed feature-extraction module is shown in Figure 5. The module is divided into two parts: (1) feature-extraction and (2) residual connection. In Figure 5, from top to bottom, the first three sets of convolutional layers are the feature-extraction parts. Set 1 consists of a 1×1 filter, a 1×3 filter, and a 1×3 filter. Set 2 consists of a 1×1 filter and a 1×3 filter, and Set 3 consists of a 1×1 filter. Moreover, Set 4 at the bottom is the residual connection part, consisting of one 1×1 filter and one 1×3 filter with a step length of 2. The multiple convolution filters enabled the model to learn multiscale features from the signal. Subsequently, the three feature vector sets were concatenated and connected through linear activation and max-pooling layers. To implement a residual connection, the output dimensionality of Set 4 was set to be the same as that of the max-pooling layer. Hence, the stride of the 1×3 filter in Set 4 was equal to 2. Finally, the output of the module was the sum of the extraction and residual parts with the ReLU activation function. When designing the feature-extraction module, the following concepts were satisfied: the 1×1 convolution filter should first be used to scale down the dimensionality before applying different kernels. The number of convolutional kernels should be larger in a more complex feature-extraction set. In Figure 5, Set 1, which had two 1×3 convolutional layers, had more kernels than the other sets. Furthermore, Set 3 only consisted of a 1×1 convolutional layer with the smallest kernel numbers. Furthermore, in Figure 5, only the filter with the purple background outputs with a linear activation function, and the other filters in the module output with the ReLU activation function.

### 3.3. Global Average Pooling

**Figure 6:**
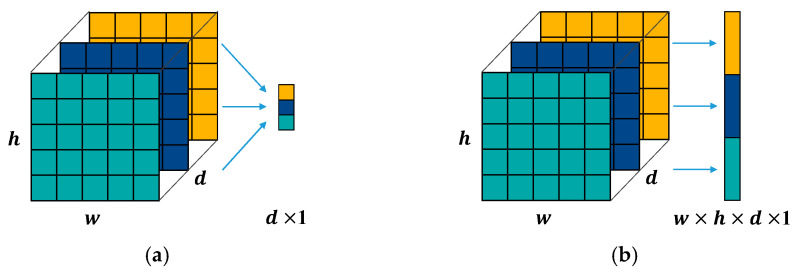
Global average pooling (GAP) and flatten layer: (**a**) GAP layer and (**b**) flatten layer.

In this study, the proposed 1D CNN replaced the flatten layer with a global average pooling (GAP) layer [39]. Figure 6 illustrates a schematic of the GAP and flatten layers. It was assumed that the feature maps obtained from the last feature-extraction module had the dimensions of width w, height h, and map number d. Classification of a dense layer with k class was connected to it. The number of parameters, $P_{g}$, required by the fully connected layer was calculated as follows:

(4) $$P_{g}=d\times k,$$

The output vector of the GAP layer represented only the spatial average of the feature map. However, the number of training parameters, $P_{f}$, required between the flatten layer and the classification layer was calculated as

(5) $$P_{f}=w\times h\times d\times k,$$

Comparing the calculation results of Equations (1) and (2) shows that the GAP layer required a fewer number of training parameters t. A lower proportion of the fully connected layer parameters to the total can decrease the chance of overfitting the problem to the training data.

**Figure 7:**
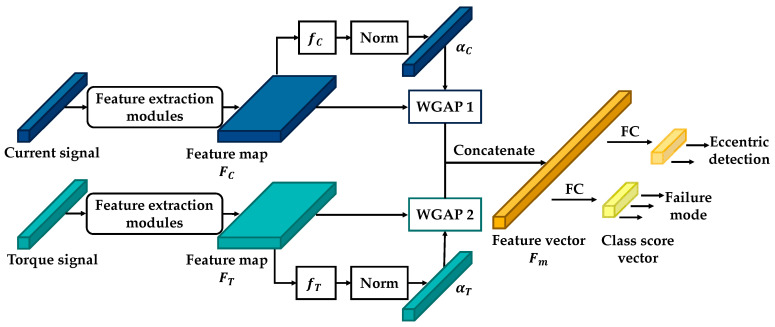
Network architecture of the proposed 1D CNN model.

Furthermore, some researchers have demonstrated that the GAP layer can be effectively utilized in 2D object localization [40,41]. In this study, weighted global average pooling (WGAP) was adopted to determine the frequency components that contributed to the signal-level classification. The network architecture of the proposed model is illustrated in Figure 7. The network is a two-input, two-output model. The two inputs are the frequency-domain signals of the current and torque. The features of the two input signals were extracted individually using several feature-extraction modules. Subsequently, the two WGAP layers were individually used to obtain the most representative features from the feature maps $F_{T}$ and $F_{c}$. Then, the two feature vectors, WGAP1 and WGAP2, were combined to obtain the fusion feature vector $F_{m}$. Finally, $F_{m}$ was used to classify the failure mode and eccentric effect. 

The last feature map obtained from the signal had a size of D×L×1. The feature map can be expressed by the following equation:

(6) $$F=\left[F_{1},\cdots ,F_{L}\right],\;F_{i}\epsilon R^{D\times 1},$$

where D and L indicate the depth and length of the feature map, respectively. Each vector $F_{i}$ is a D-dimensional vector representing the feature of the spatial region i. The output vector of the WGAP layer, $G\epsilon R^{D\times 1}$, is expressed as

(7) $$G=\left[\sum_{i=1}^{L}\alpha_{i}F_{i,1},\;\cdots ,\;\sum_{i=1}^{L}\alpha_{i}F_{i,D}\right],$$

where $\alpha \epsilon R^{L\times 1}$ is the weight matrix. The weight matrix evaluates the critical grade of the spatial ith region. The value of the weights is normalized to the range [0,1], and the sum of the weight values is equal to 1. Generally, a spatial region with a higher weight implies that it is more informative for signal-level classification. The weight matrix was determined as follows:

(8) $$\alpha =\left[\frac{\exp\left(f_{1}\right)}{\sum_{j=1}^{L}\exp\left(f_{j}\right)},\;\cdots ,\;\frac{\exp\left(f_{L}\right)}{\sum_{j=1}^{L}\exp\left(f_{j}\right)}\right],$$

where $f\epsilon R^{L\times 1}$, which is the feature score of the ith region, is obtained by the following operation:

(9) $$f=\left[\sigma \left(WF_{1}+b\right),\;\cdots ,\sigma \left(WF_{L}+b\right)\right],$$

where $W\epsilon R^{1\times D}$ is the parameter vector and $b\epsilon R^{1\times 1}$ is the bias. σ is the tanh activation function. By concatenating the two outputs of the WGAP1 and WGAP2 layers, the fusion feature vector $F_{m}\epsilon R^{2D\times 1}$, shown in Figure 7, is obtained. Then, two FC layers with a softmax activation function are used separately to classify the fusion feature vector $F_{c}$. For a given class c, the probability distribution of classification $P_{c}$ is expressed as follows:

(10) $$P_{c}=\frac{\exp\left(S_{c}\right)}{\sum_{i=1}^{c}\exp\left(S_{i}\right)},$$

where $S_{c}$ is the input vector of the softmax layer, which is determined using the following equation:

(11) $$S_{c}=\overset{2D}{\underset{n=1}{\sum }}w_{n}^{c}F_{m,n},$$

where $w_{n}^{c}$ is the classification weight corresponding to class c for unit n. To visualize and analyze the important frequency component for a particular signal, a class feature map (CFM) was built. The CFMs for different signals are defined as follows:

(12) $$\{\begin{array}{c} M_{C,c}=\overset{D}{\underset{n=1}{\sum }}(0.5w_{n}^{c,1}F_{C,n}+0.5w_{n}^{c,2}F_{C,n})\\ M_{T,c}=\overset{2D}{\underset{n=D+1}{\sum }}(0.5w_{n}^{c,1}F_{T,n}+0.5w_{n}^{c,2}F_{T,n}) \end{array},$$

where $M_{C,c}$ and $M_{T,c}$ are the CFMs corresponding to class c of the current signal and the torque signal, respectively. CFM is the linear sum of the patterns at different spatial locations. $w_{n}^{c,1}$ and $w_{n}^{c,2}$ indicate the classification weights of the two types of outputs. A constant value of 0.5 indicates that $w_{n}^{c,1}$ and $w_{n}^{c,2}$ equally influence the result of the CFM. To observe the critical grade of the frequency component, the CMF should be scaled to the size of the corresponding original signal, and the amplitude of the CMF should be normalized from 0 to 1. Finally, the users can identify the frequency regions that are most relevant to a particular category.

### 3.4. Model Building

**Figure 8:**
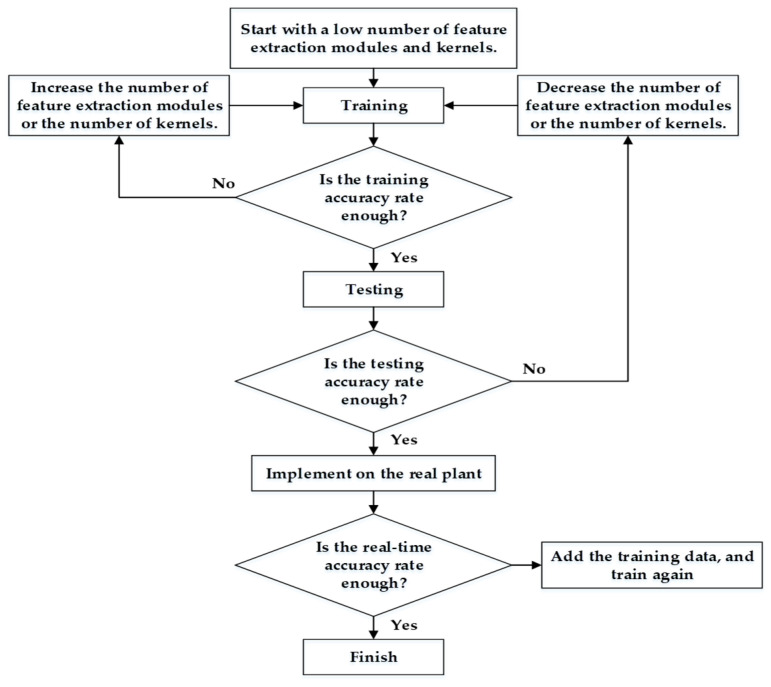
Flow chart of the diagnosis model design process.

To confirm that the proposed 1D CNN model could be implemented on a real platform, a simple design flowchart was designed and is presented in Figure 8. The principles used to adjust the parameters are described in Section 3.2. For the feature-extraction module, the kernel number of Set 3 must exceed that of Set 2, and the kernel number of Set 3 must be the least. 

To ensure that the random parameters did not construct the final neural network, the neural network was built from a comparatively small size. If the average training accuracy is lower than 90%, users need to add more feature-extraction modules and follow the rules presented in Figure 8. Furthermore, once the average training accuracy is sufficiently high, users should check whether the difference between the average training and average testing accuracies is lower than 3%, implying that the overfitting problem is not significant. Subsequently, the model will be tested on a real platform. To verify that the model can be implemented on the motor diagnosis platform, 10 continuous predictions were used to determine the motor state for one diagnostic result. The failure mode with the highest number among the 10 predictions was the diagnostic result. The eccentric mode was diagnosed using the same method.

The architecture of the proposed 1D CNN model after experimental tuning is shown in Figure 7. Generally, the architecture of the proposed model has two input layers: one for the current signal and the other for the torque signal. For both input signals, seven feature-extraction modules and one WGAP layer were used to learn the features. Subsequently, one concatenated layer and two output layers were connected separately. To avoid the overfitting problem and generalize the ability of the 1D CNN model, the dropout regularization method [42] was used between the WGAP layer and the output layer with a rate of 0.2. Table 2 and Table 3 show the hyperparameter settings of the proposed motor diagnosis model and feature-extraction module, respectively. 

The proposed 1D CNN model was trained using the Adam optimization [43] with the mean square error function. Based on a large number of experiments and observation of the classification accuracy, a fixed learning rate of 3 × 10^−4^ was assigned. Finally, the softmax function was used to classify the feature vector into three failure modes and eccentric detection. The proposed 1D CNN model performs a high classification accuracy rate. The experimental results and analysis are presented and discussed in Section 4.

## 4. Experimental Results

In this section, the results of the experiment are discussed. First, the formula for calculating the accuracy rate is presented. Then, the evaluation of the performance of the proposed 1D CNN model and the comparison with other algorithms, including KNN, SVM, multilayer perceptron (MLP), 1D CNNG, 1D CNNT, and 1D CNNC, are discussed. In addition, the t-SNE [44] algorithm is used to reduce the dimensionality of the feature map and visualize the separation of the features learned from the model. Then, the relevant frequency components obtained automatically by the proposed 1D CNN are analyzed.

### 4.1. Classification Results

To evaluate the proposed 1D CNN model, the following formulas were used to calculate the classification accuracy:

(13) $$Acc_{mode}=\frac{T_{hm}+T_{dm}+T_{bm}}{T_{hm}+T_{dm}+T_{bm}+F_{hm}+F_{dm}+F_{bm}}\times 100\%$$

(14) $$Acc_{ecc}=\frac{T_{ne}+T_{e}}{T_{ne}+T_{e}+F_{ne}+F_{e}}\times 100\%$$

(15) $$Acc_{state}=\frac{T_{state}}{T_{state}+F_{state}}\times 100\%$$

(16) $$Acc_{avg}=\frac{Acc_{mode}+Acc_{ecc}}{2}\times 100\%$$

where $Acc_{mode}$ and $Acc_{ecc}$ denote the classification accuracies of the failure mode and eccentricity, respectively. $T_{hm}$, $T_{dm}$, and $T_{bm}$ indicate the true classifications of the healthy, demagnetized, and bearing fault motors, respectively. $T_{ne}$ and $T_{e}$ represent the conditions without and with an eccentric effect, respectively. $F_{hm}$, $F_{dm}$, $F_{bm}$, $F_{ne}$, and $F_{e}$ are the corresponding false classifications. In addition, the accuracy rate of the individual state, $Acc_{state}$, was calculated using Equation (15), where $T_{state}$ and $F_{state}$ represent the true and false detections, respectively. $Acc_{avg}$ indicates the average accuracy of the method. Both the classification of the failure modes and the eccentricity are equally important; hence, the denominator of Equation (16) is 2.

The following methods were evaluated for comparison.

1. SVM using the handcrafted features;
2. KNN classifier using the handcrafted features;
3. MLP: a two-input, two-output model;
4. 1D CNNG: a two-input, two-output model based on [29,30], and that uses current and torque signals;
5. 1D CNNC: a one-input, two-output model based on the proposed method, but that only uses current signal;
6. 1D CNNT: a one-input, two-output model based on the proposed method, but that only uses torque signal;
7. Proposed 1D CNN using current and torque signals.

Two classical machine-learning methods, KNN and SVM, were used. The KNN and SVM methods were trained using handcrafted features. The handcrafted features used are listed in Table 4. The current and torque signals were analyzed using the DWT [29], considering the seventh-level decomposition. The Daubechies 24 was used as the mother wavelet function. For each level, features 1–6 in Table 4 were extracted. To solve the confusion caused by the similar failure modes under different magnitudes of loads, the maximum amplitude of the time-domain and frequency-domain signals were extracted (features 7 and 8) as well. Therefore, the feature vector for KNN and SVM was 100 (8 × 6 + 2 for each signal). In this study, the KNN used the KD-tree algorithm to compute the nearest neighbors, and the number of neighbors was five. In the SVM, both the linear and RBF functions were used as the kernel functions for evaluation.

For the remaining learning methods, models with a similar number of parameters (approximately 170,000) were designed for the analysis and comparison. MLP, 1D CNNG, and the proposed 1D CNN models used the normalized amplitude–frequency signals of the current and torque as the input signals. To confirm the effectiveness of the fusion features, the performance of the 1D CNNC and 1D CNNT models, which use a single signal only as the input vector, were evaluated. For the MLP method, two processing streams consisting of one fully connected layer with 14 neurons were used to learn features from the signals separately. The 1D CNNG model was constructed according to [29,30] and consisted of two input layers, four densely connected convolutional layers, a flatten layer, and two output layers. In 1D CNNG, the kernel size went from large to small, and the number of kernels increased as the model went deeper. As for the 1D CNNC and 1-D CNNT, both models consisted of one input layer, seven feature-extraction modules, a WGAP layer, and two output layers. The classification results obtained from the comparison are listed in Table 5.

Table 5 shows the classification accuracies of the individual states and the average accuracies. The average classification accuracies of KNN and SVM based on the handcrafted features were 88.96% and 94.66%, respectively. The classification accuracy of KNN and SVM was up to 85.00%; however, it was still lower than that of the proposed 1D CNN model. For such a complex dataset, handcrafted features cannot comprehensively represent the data. However, learning methods can effectively learn discriminated features from input signals. For the MLP and 1D CNNG methods, the average accuracies were 89.81% and 93.36%, respectively. Compared with the result of MLP, the architecture of the 1D CNN was more suitable for the frequency-domain signals used in this study. The 1D CNNG model performed well in the failure mode classification with accuracies of 100%, 99.89%, and 99.18% for the healthy, bearing fault, and demagnetized motors, respectively. However, the 1D CNNG model could not effectively determine whether the eccentric phenomenon occurred in the mechanical system—the accuracy rates were 99.60% and 74.49%.

Furthermore, based on the experimental results, the stacking of the densely connected convolutional layers and flatten layer used in 1D CNNG caused an overfitting problem. In the training stage, the experimental results revealed that the failure mode and eccentricity detection accuracies of 1D CNNG were up to 99.00% and 97.20%, respectively. The average accuracy was 98.1%. However, in the testing stage, the accuracy rates of the motor failure mode and eccentricity classifications were 99.69% and 87.04%, respectively. Moreover, the average accuracy rate was 93.36%, which was relatively lower than 98.1%. The overfitting problem was solved by replacing the densely connected convolutional layers and the flatten layer with the proposed feature-extraction modules and WGAP layers. Based on the experimental results, the average accuracy rates of 1D CNNT, 1D CNNC, and proposed 1D CNN in the training stage were 90.04%, 95.05%, and 99.43%, respectively. In the testing stage, the average accuracy rates of the 1D CNNT, 1D CNNC, and proposed 1D CNN were 89.80%, 94.80%, and 98.85%, respectively, which were close to the results in the training stage. The results show that the reduction of training parameters for the fully connected layer between the feature vector and the output layers could lower the possibility of overfitting.

Furthermore, a comparison between the 1D CNNT, 1D CNNC, and the proposed 1D CNN models is discussed. Table 5 indicates that the accuracy rate of the proposed 1D CNN model was higher than those of the similar models using the single sensor information, which confirmed the effectiveness of the fusion features learned from the extraction modules. The average accuracy of the proposed model was 98.85%, which was 4.5%, and 9.5% higher than those of the 1D CNNC and 1D CNNT, respectively. For the CNNT model, the accuracies of the failure mode and eccentricity detection were 90.41% and 89.20%, respectively. Moreover, that of the 1D CNNC model were 98.93% and 90.675%, respectively. Both models had an approximately 90% accuracy rate of eccentricity detection. However, when using a multi-signal network, the accuracy was improved to 98.04%. As for the failure mode detection, CNNC already achieved a 98.93% accuracy rate. After combining the current and torque signal information, the accuracy rate increased to 99.66%.

**Figure 9:**
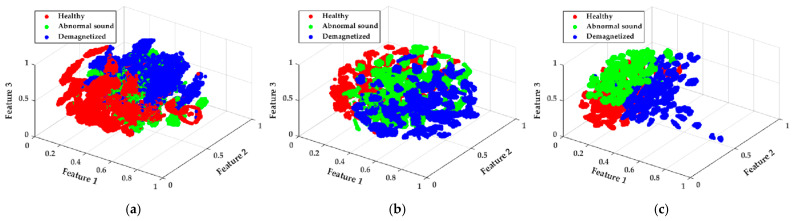
Visualization of the low-dimensional feature vector: (**a**) 1-D CNNT, (**b**) 1-D CNNC, and (**c**) the proposed model.

To provide an intuitive understanding of the effectiveness of the proposed method, the feature vectors learned from CNNT, CNNC, and the proposed model were visualized using the t-SNE algorithm. The t-SNE algorithm is a technique for dimensionality reduction. The 248-dimensional feature vector was reduced to three, and the visualization results are shown in Figure 9. By rotating and observing the plot, the features can roughly separate the three classes of failure modes. However, Figure 9a shows that the features of the three failure modes were easily clustered at the boundaries between each class. The separation of the three failure modes is better in Figure 9b than in Figure 9a; however, the features of the healthy, bearing fault, and demagnetized motor are sparsely separated into several groups. In Figure 9c, most features are distributed on the left side of the plot. However, it can be observed that the clustering of features at the boundary was not critical, as shown in Figure 9a. Furthermore, similar to Figure 9b, the features of the three failure modes were separated into several groups, but each group was closer than that shown in Figure 9b. The results shown in Table 5 and Figure 8 confirm the effectiveness of the proposed feature-extraction module and 1D CNN on multisensory fusion.

### 4.2. Important Frequency Component

**Figure 10:**
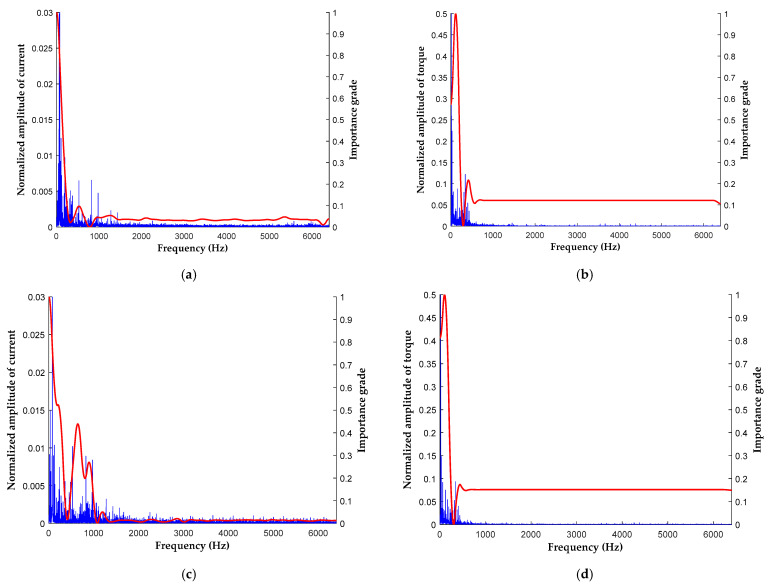
Important grade of the frequency component conducted under the 1000 rpm, 0.24 Nm, and no eccentric effect operating conditions: (**a**) current signal of the motor with bearing fault, (**b**) torque signal of the motor with bearing fault, (**c**) current signal of the demagnetized motor, and (**d**) torque signal of the demagnetized motor.

To find the important frequencies contributing to specific classifications, the CMF was generated and visualized in this section. Figure 10 shows the important grades of the frequency components for different failure modes under similar operating conditions. The evaluated operating conditions were under 1000 rpm, 0.24 Nm loading, and no eccentric effect. 

Comparing Figure 10a with Figure 10c, the current frequency spectra and the curves of the important grade clearly demonstrate the difference between the two types of motors. However, the torque frequency spectra and the curves of the important grade for both motors were similar. As shown in Figure 10a,c, frequency components smaller than 400 Hz had similar important grades for deciding the classification. Nevertheless, the model took more important grades in the range of 400–1050 Hz to obtain the classification results of the demagnetized motor. In Figure 10a, the peak value is located at approximately 550 Hz, whereas in Figure 10c, the two peak values are located at approximately 650 and 900 Hz. Furthermore, in Figure 10b,d, the two peak values are located at approximately 120 Hz and 425 Hz, respectively. However, in Figure 10d, frequency components smaller than 120 Hz accounted for a larger important grade, and the frequency amplitude at 425 Hz was larger than that in Figure 10b. The analyses in Section 4.2 and Section 4.3 indicate that the current frequency spectrum mainly determined the classification results. Furthermore, the model could achieve a higher classification accuracy by combining the torque frequency information in the two influential regions.

### 4.3. Training Time and Prediction Speed

Table 6 lists the training and prediction speeds of each model. The prediction time in Table 6 is the average time of 1000 prediction times. For the classical machine-learning methods, the training times of the KNN and SVM were 0.09 and 9.32 min, respectively. The training times were significantly shorter than those of the deep-learning methods. However, the training times do not account for the time required for feature-extraction. It took about 6 h to extract the handcrafted features from the entire datasets. The deep-learning method could simultaneously perform feature-extraction during training. The training times of the MLP, 1D CNNG, 1D CNNT, 1D CNNC, and proposed method were 33.28, 127.92, 120.93, and 85.06 min, respectively. Although the MLP and 1D CNNG methods required less time to train the model, their performances were typically inferior. By combining the features of the current and torque signals, the proposed model could converge faster, and took less time for training than the models that used a single signal. 

The SVM could perform the fastest prediction. The proposed method took 0.0335 s to predict a single sample, whereas the other models took approximately 0.025 s to predict one sample. The prediction time of the proposed method was ten times longer than that of the SVM. However, on the real platform, we used 10 continuous predictions to determine one diagnostic result. Hence, it took approximately 0.335 s to diagnose the state of the motor each time for the proposed model; 0.335 s is sufficiently short for the motor diagnosis system. Furthermore, among the deep-learning methods, the proposed model had the highest performance. 

### 4.4. Computation System

The computer used for training in this work was a PC equipped with an NVIDIA GeForce GTX 1080Ti and an Intel^®^ Core™ I7-8700K. The PC had a 32G memory to calculate such a large dataset. This work used Python as a development tool owing to its convenient libraries, such as Scikit-Learn, Keras, and TensorFlow. The drawings in this article were done in MATLAB, owing to the convenient drawing functions and esthetics of its figures.

## 5. Conclusions

### 5.1. Discussion of Results

This paper proposes a 1D CNN model, which is a multi-signal fault diagnosis network for PMSMs. The experimental results reveal that the current and torque signal fusion features enable the model to perform better than single-signal models. The operating conditions evaluated included a wide speed range (100–1600 rpm), loading effect (0–0.24 Nm), and eccentric effect. The variable speeds, loads, and eccentricity resulted in a detection more similar to the actual applications. For humans, such complex operating conditions are difficult to classify and require considerable time for analysis. By using the handcrafted features, the KNN and SVM classifiers achieved classification accuracies of 88.96% and 94.66%, respectively. However, the designed 1D CNN model could automatically find the discriminated features under such complex conditions, and effectively classified three failure modes and eccentricity with accuracies of 99.66% and 98.04%, respectively. The total accuracy rate was up to 98.85%. Compared with a previous study, the dataset used in this study was relatively larger. The overfitting phenomenon was not significant.

Furthermore, this research extended the technique of 2D image object localization to a 1D signal important segment finding. The proposed model with the WGAP layer could generate the CMFs of the current and torque signals. CMF could effectively identify the important frequency components contributing to the classification. By observing the CMFs under different operating conditions, users can determine the difference in signals between normal and failure motors. 

### 5.2. Future Work

We expect the motor fault diagnosis system to be used in the status monitoring of production lines. However, the system proposed in this paper is based on PC; the equipment cost and electricity consumption for a PC-based system are high. In the future, we will only use the PC to develop and design the detection model, and the embedded system will be used to implement the diagnosis. We intend to modify the proposed diagnosis system from a PC to an embedded board, such as the Jetson Nano. By observing the CMFs, the unimportant frequency components can be discarded and the size of the input vectors can be reduced. Moreover, by changing the computation platform and data acquisition unit, the cost of implementing the diagnosis to the production line can be reduced. In addition, the 1×1 kernel and the WGAP layer used in the proposed model can decrease the size of the model. These advantages can lower the computational resources and make the model more suitable for the embedded systems. 

## Figures and Tables

**Table 1:** Types of motor conditions.

Index	Motor Type	Operation Condition
1	Healthy motor	Loading conditions: 0 Nm, 0.24 Nm, and 0–0.24 Nm
2	Demagnetized motor	Rotating speeds: 100–1600 rpm
3	Motor with bearing fault	Eccentricity: Yes and no

**Table 2:** Parameter settings of the proposed motor diagnosis model.

Size of the input signal	6400 × 1
Input size of the 1st feature-extraction module	6400 × 1
Input size of the 2nd feature-extraction module	3200 × 6
Input size of the 3rd feature-extraction module	1600 × 12
Input size of the 4th feature-extraction module	800 × 12
Input size of the 5th feature-extraction module	400 × 29
Input size of the 6th feature-extraction module	200 × 29
Input size of the 7th feature-extraction module	100 × 93
Input size of the WGAP layer	50 × 124
Input size of the concatenate layer: (1) WGAP 1 current signal	124
(2) WGAP 2 torque signal	124
Dropout layer 1 and dropout layer 2	
Input size of the output layer 1	248
Input size of the output layer 2	248
The output size of the output layer 1 (failure mode classification)	3
The output size of the output layer 2 (eccentricity detection)	2
Number of samples for training	115,200
Number of samples for testing	28,800
Learning rate	3 × 10^−4^
Dropout ratio	0.2

**Table 3:** Hyperparameter settings of each feature-extraction module.

Index of the Feature Extraction Module	Number of Kernel							
	Set 1			Set 2		Set 3	Set 4	
	1 × 1	1 × 3	1 × 3	1 × 1	1 × 3	1 × 1	1 × 1	1 × 3
1	1	2	3	1	2	1	2	6
2	2	4	6	2	4	2	4	12
3	2	4	6	2	4	2	4	12
4	5	10	15	4	10	4	10	29
5	5	10	15	4	10	4	10	29
6	15	30	48	15	30	15	31	98
7	24	32	64	24	36	24	41	124

**Table 4:** Handcrafted feature sets for k-nearest neighbor (KNN) and support vector machine (SVM).

Index	Features	Formations
1	Mean	$\mu_{X}=\frac{1}{N}\overset{N}{\underset{i=1}{\sum }}X_{i}$
2	Median	$Med=\frac{X\left[\frac{n-1}{2}\right]+X\left[\frac{n+1}{2}\right]}{2}$
3	Standard deviation	$\sigma =\sqrt{\frac{1}{N}\overset{N}{\underset{i=1}{\sum }}{\left(X_{i}-\mu_{X}\right)}^{2}}$
4	Median absolute deviation	$Med_{AD}=median\left(\left|X_{i}-median\left(X\right)\right|\right)$
5	Mean absolute deviation	$Mean_{AD}=\frac{1}{N}\overset{N}{\underset{i=1}{\sum }}\left|X_{i}-\mu_{X}\right|$
6	Percentage of energy	$E_{l}=\overset{N}{\underset{j=1}{\sum }}\frac{X_{lj}^{2}}{\sum_{i=1}^{8}\sum_{j=1}^{N}X_{ij}^{2}}$
7	The maximum amplitude of the time-domain signal	$M_{t}=max\left(S_{t}\right)$
8	The maximum amplitude of the frequency-domain signal	$M_{f}=max\left(S_{f}\right)$

**Table 5:** Diagnosis accuracy rate of the methods.

Method	Accuracy Rate					
	Healthy	Bearing Fault	Demagnetized	with No Eccentricity	with Eccentricity	Average
KNN	98.12%	94.44%	90.45%	86.57%	80.60%	88.96%
SVM	99.72%	99.96%	99.72%	89.23%	89.65%	94.66%
MLP	95.12%	88.78%	96.18%	88.02%	84.50%	89.81%
1D CNNG	100.00%	99.89%	99.18%	99.60%	74.49%	93.36%
1D CNNT	94.73%	87.58%	88.93%	87.89%	90.52%	89.80%
1D CNNC	99.68%	98.78%	98.33%	92.28%	89.07%	94.80%
Proposed 1D CNN	99.89%	99.90%	99.19%	97.59%	98.49%	98.85%

**Table 6:** Training time and prediction speeds of different models.

Models	Training Time (min)	Prediction Time (s)
KNN	0.09	0.0210
SVM	9.32	0.0045
MLP	16.28	0.0235
1D CNNG	33.28	0.0254
1D CNNT	127.92	0.0266
1D CNNC	120.93	0.0263
Proposed 1D CNN	85.06	0.0335

## Data Availability

Not applicable.

## References

[1] Lei Y., Jia F., Lin J., Xing S., Ding S.X. (2016). An Intelligent Fault Diagnosis Method Using Unsupervised Feature Learning towards Mechanical Big Data. IEEE Trans. Ind. Electron..

[2] Gangsar P., Tiwari R. (2020). Signal based condition monitoring techniques for fault detection and diagnosis of induction motors: A state-of-the-art review. Mech. Syst. Signal Process..

[3] Liang X., Ali M.Z., Zhang H. (2020). Induction Motors Fault Diagnosis Using Finite Element Method: A Review. IEEE Trans. Ind. Appl..

[4] Yassa N., Rachek M., Houassine H. (2019). Motor Current Signature Analysis for the Air Gap Eccentricity Detection in the Squirrel Cage Induction Machines. Energy Procedia.

[5] Abdelkrim C., Meridjet M.S., Boutasseta N., Boulanouar L. (2019). Detection and classification of bearing faults in industrial geared motors using temporal features and adaptive neuro-fuzzy inference system. Heliyon.

[6] Li C., Xiong J., Zhu X., Zhang Q., Wang S. (2020). Fault Diagnosis Method Based on Encoding Time Series and Convolutional Neural Network. IEEE Access.

[7] Zaman S.M.K., Liang X. (2021). An Effective Induction Motor Fault Diagnosis Approach Using Graph-Based Semi-Supervised Learning. IEEE Access.

[8] Jafari A., Faiz J., Jarrahi M.A. (2021). A simple and efficient current-based method for inter-turn fault detection in BLDC motors. IEEE Trans. Ind. Inform..

[9] Shifat T.A., Hur J.-W. (2021). ANN assisted multi sensor information fusion for BLDC motor fault diagnosis. IEEE Access.

[10] Cheng L., Tian G.Y. (2011). Surface Crack Detection for Carbon Fiber Reinforced Plastic (CFRP) Materials Using Pulsed Eddy Current Thermography. IEEE Sens. J..

[11] Kou L., Qin Y., Zhao X., Chen X. (2019). A Multi-Dimension End-to-End CNN Model for Rotating Devices Fault Diagnosis on High-Speed Train Bogie. IEEE Trans. Veh. Technol..

[12] Luo B., Wang H., Liu H., Li B., Peng F. (2019). Early Fault Detection of Machine Tools Based on Deep Learning and Dynamic Identification. IEEE Trans. Ind. Electron..

[13] Glowacz A., Glowacz Z. (2017). Diagnosis of the three-phase induction motor using thermal imaging. Infrared Phys. Technol..

[14] Glowacz A. (2018). Acoustic based fault diagnosis of three-phase induction motor. Appl. Acoust..

[15] Glowacz A., Glowacz W., Glowacz Z., Kozik J. (2018). Early fault diagnosis of bearing and stator faults of the single-phase in-duction motor using acoustic signals. Measurement.

[16] Nivesrangsan P., Jantarajirojkul D. Bearing fault monitoring by comparison with main bearing frequency components using vibration signal. Proceedings of the 2018 5th International Conference on Business and Industrial Research (ICBIR).

[17] Liu Y., Qiao N., Zhao C., Zhuang J. (2018). Vibration Signal Prediction of Gearbox in High-Speed Train Based on Monitoring Data. IEEE Access.

[18] Zhang Z., Verma A., Kusiak A. (2012). Fault Analysis and Condition Monitoring of the Wind Turbine Gearbox. IEEE Trans. Energy Convers..

[19] Chen X., Feng Z. (2017). Time-Frequency Analysis of Torsional Vibration Signals in Resonance Region for Planetary Gearbox Fault Diagnosis Under Variable Speed Conditions. IEEE Access.

[20] Bravo-Imaz I., Ardakani H.D., Liu Z., García-Arribas A., Arnaiz A., Lee J. (2017). Motor current signature analysis for gearbox condition monitoring under transient speeds using wavelet analysis and dual-level time synchronous averaging. Mech. Syst. Signal Process..

[21] Azamfar M., Singh J., Bravo-Imaz I., Lee J. (2020). Multisensor data fusion for gearbox fault diagnosis using 2-D convolutional neural network and motor current signature analysis. Mech. Syst. Signal Process..

[22] Giantomassi A., Ferracuti F., Iarlori S., Ippoliti G., Longhi S. (2015). Electric Motor Fault Detection and Diagnosis by Kernel Density Estimation and Kullback–Leibler Divergence Based on Stator Current Measurements. IEEE Trans. Ind. Electron..

[23] Dias C.G., Pereira F.H. (2018). Broken rotor bars detection in induction motors running at very low slip using a hall effect sensor. IEEE Sens. J..

[24] Liu H., Zhou J., Xu Y., Zheng Y., Peng X., Jiang W. (2018). Unsupervised fault diagnosis of rolling bearings using a deep neural network based on generative adversarial networks. Neurocomputing.

[25] Altobi M.A.S., Bevan G., Wallace P., Harrison D., Ramachandran K. (2019). Fault diagnosis of a centrifugal pump using MLP-GABP and SVM with CWT. Eng. Sci. Technol. Int. J..

[26] Moosavi S.S., Djerdir A., Amirat Y.A., Khaburi D.A. (2015). ANN based fault diagnosis of permanent magnet synchronous motor under stator winding shorted turn. Electr. Power Syst. Res..

[27] Ahmad W., Khan S.A., Kim J.-M. (2018). A Hybrid Prognostics Technique for Rolling Element Bearings Using Adaptive Predictive Models. IEEE Trans. Ind. Electron..

[28] Ali M.Z., Shabbir M.N.S.K., Liang X., Zhang Y., Hu T. (2019). Machine learning-based fault diagnosis for single- and multi-faults in induction motors using measured stator currents and vibration signals. IEEE Trans. Ind. Appl..

[29] Kao I.-H., Wang W.-J., Lai Y.-H., Perng J.-W. (2018). Analysis of Permanent Magnet Synchronous Motor Fault Diagnosis Based on Learning. IEEE Trans. Instrum. Meas..

[30] Shao S., Yan R., Lu Y., Wang P., Gao R.X. (2019). DCNN-Based Multi-Signal Induction Motor Fault Diagnosis. IEEE Trans. Instrum. Meas..

[31] Yao D., Liu H., Yang J., Li X. (2020). A lightweight neural network with strong robustness for bearing fault diagnosis. Measurement.

[32] Kumar A., Vashishtha G., Gandhi C.P., Zhou Y., Glowacz A., Xiang J. (2021). Novel Convolutional Neural Network (NCNN) for the Diagnosis of Bearing Defects in Rotary Machinery. IEEE Trans. Instrum. Meas..

[33] Wang J., Fu P., Zhang L., Gao R.X., Zhao R. (2019). Multilevel Information Fusion for Induction Motor Fault Diagnosis. IEEE/ASME Trans. Mechatron..

[34] Szegedy C., Liu W., Jia Y., Sermanet P., Reed S., Anguelov D., Erhan D., Vanhoucke V., Rabinovich A. (2014). Going Deeper with Convolutions. arXiv.

[35] Szegedy C., Vanhoucke V., Ioffe S., Shlens J., Wojna Z. (2015). Rethinking the inception architecture for computer vision. arXiv.

[36] He K., Zhang X., Ren S., Sun J. Deep residual learning for image recognition. Proceedings of the IEEE Conference on Computer Vision and Pattern Recognition.

[37] Szegedy C., Ioffe S., Vanhoucke V., Alemi A.A. Inception-v4, inception-ResNet and the impact of residual connections on learning. Proceedings of the Thirty-First AAAI Conference on Artificial Intelligence.

[38] Zhang R., Bahrami Z., Wang T., Liu Z. (2021). An Adaptive Deep Learning Framework for Shipping Container Code Localization and Recognition. IEEE Trans. Instrum. Meas..

[39] Gong W., Chen H., Zhang Z., Zhang M., Gao H. (2020). A Data-Driven-Based Fault Diagnosis Approach for Electrical Power DC-DC Inverter by Using Modified Convolutional Neural Network with Global Average Pooling and 2-D Feature Image. IEEE Access.

[40] Zhou B., Khosla A., Lapedriza A., Oliva A., Torralba A. Learning Deep Features for Discriminative Localization. Proceedings of the 2016 IEEE Conference on Computer Vision and Pattern Recognition.

[41] Qiu S. (2018). Global weighted average pooling Bbridges pixel-level localization andimage-level classification. arXiv.

[42] Krizhevsky A., Sutskever I., Hinton G.E. (2012). Imagenet classification with deep convolutional neural networks. Commun. ACM.

[43] Kingma D.P., Ba J. (2017). Adam: A method for stochastic optimization. arXiv.

[44] van der Maaten L., Hinton G. (2008). Visualizing data using t-SNE. J. Mach. Learn. Res..

